# New Avenues and Major Achievements in Phytocompounds Research for Glioblastoma Therapy

**DOI:** 10.3390/molecules29071682

**Published:** 2024-04-08

**Authors:** Aleksandra Majchrzak-Celińska, Elżbieta Studzińska-Sroka

**Affiliations:** 1 Department of Pharmaceutical Biochemistry, Poznan University of Medical Sciences, Rokietnicka 3 Str., 60-806 Poznan, Poland; 2 Department of Pharmacognosy and Biomaterials, Poznan University of Medical Sciences, Rokietnicka 3 Str., 60-806 Poznan, Poland; elastudzinska@ump.edu.pl

**Keywords:** phytocompounds, glioblastoma, chemosensitization, phyto-nanoformulations, clinical trials

## Abstract

Phytocompounds have been evaluated for their anti-glioblastoma actions for decades, with promising results from preclinical studies but only limited translation into clinics. Indeed, by targeting multiple signaling pathways deregulated in cancer, they often show high efficacy in the in vitro studies, but their poor bioavailability, low tumor accumulation, and rapid clearance compromise their efficacy in vivo. Here, we present the new avenues in phytocompound research for the improvement of glioblastoma therapy, including the ways to enhance the response to temozolomide using phytochemicals, the current focus on phytocompound-based immunotherapy, or the use of phytocompounds as photosensitizers in photodynamic therapy. Moreover, we present new, intensively evaluated approaches, such as chemical modifications of phytochemicals or encapsulation into numerous types of nanoformulations, to improve their bioavailability and delivery to the brain. Finally, we present the clinical trials evaluating the role of phytocompounds or phytocompound-derived drugs in glioblastoma therapy and the less studied phytocompounds or plant extracts that have only recently been found to possess promising anti-glioblastoma properties. Overall, recent advancements in phytocompound research are encouraging; however, only with more 3D glioblastoma models, in vivo studies, and clinical trials it is possible to upgrade the role of phytocompounds in glioblastoma treatment to a satisfactory level.

## 1. Introduction

Glioblastoma (GBM) is the most common and aggressive type of primary brain tumor, with an annual incidence of 3.5 cases per 100,000 population [1]. Unfortunately, the prognosis for GBM patients has not changed significantly over the past decade, with a median overall survival (OS) ranging from 14.6 to 20.5 months and a five-year survival rate of only 5% [1,2]. The current standard of care includes surgery, radiotherapy, and chemotherapy with concomitant temozolomide (TMZ). However new therapeutic approaches are being evaluated. Those include, among others, targeted molecular therapies, autologous dendritic cell vaccines, and autologous tumor vaccines; however, they show limited success [3]. In fact, there is no consistent evidence for the survival efficacy of any personalized therapy of GBM [3]. The molecular mechanisms of treatment resistance are numerous. The highly infiltrative nature of GBM makes complete resection at the cellular level nearly impossible [4]. Abundant hypoxic regions provide perivascular niches for glioma stem cells (GSCs), which can yield potentially more aggressive recurrent tumors [4]. Moreover, the blood–brain barrier (BBB) and the brain–tumor barrier (BTB) limit the penetration of antineoplastic drugs into the brain [5]. Additionally, ATP-binding cassette transporters actively mediate the efflux of therapeutic agents out of the brain parenchyma, resulting in poor response to treatments [6,7]. Other therapeutic challenges include abundant intra- and inter-tumoral heterogeneity, dysregulated signaling pathways, and an immunosuppressive microenvironment [8,9]. Thus, novel, powerful, yet safe treatment options are urgently needed.

**Figure 1:**
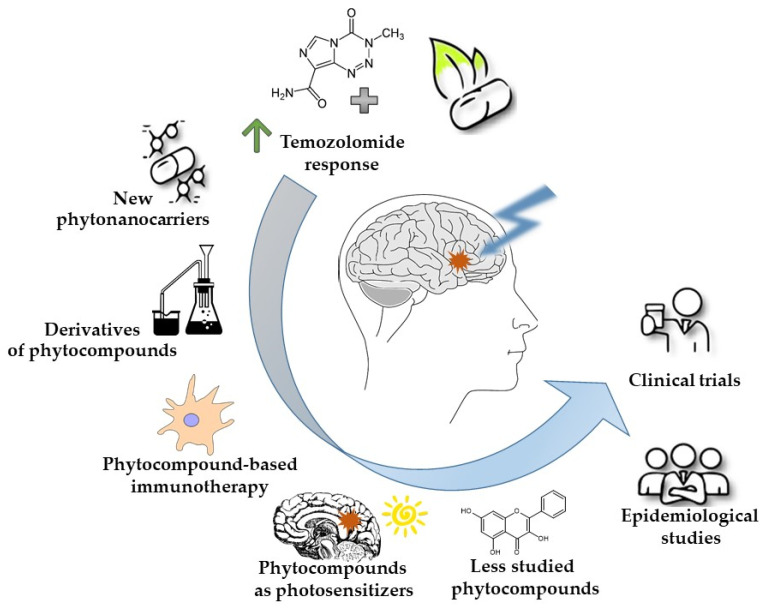
Overview of the new avenues in phytocompounds research for GBM therapy. The major trends include increasing the effectiveness of classical chemotherapy, developing new phyto-nanocarriers, producing chemical derivatives of phytocompounds, using phytocompounds as immunomodulatory agents or photosensitizers in photodynamic therapy, deciphering the role of less studied compounds, and eventually, performing epidemiological studies and clinical trials.

The idea of targeting GBM using phytocompounds (i.e., secondary metabolites of plants) has a long history. In this regard, several compounds, i.e., curcumin, isothiocyanates, resveratrol, and epigallocatechin-3-gallate (EGCG), have been evaluated in numerous studies, showing activity against GBM cells. However, the implementation of the phytocompound laboratory research into clinical reality is still unsatisfactory. Poor bioavailability, low tumor accumulation, and rapid clearance in vivo compromise the efficacy of phytocompounds in clinical trials. Thus, new ideas are being developed, and new approaches are being evaluated in order to upgrade the phytotherapy of GBM to a satisfactory level (Figure 1). In this context, the innovative phytotherapy of GBM includes the development of BBB-penetrating agents, such as nanoformulations of active phytochemicals. Research shows that the solubility, stability, and pharmacokinetic and pharmacodynamic characteristics of phytocompounds can be improved with the use of nanoformulations [10]. Moreover, chemical modifications of phytochemicals to improve their bioavailability have also been developed and tested, with promising results. Additionally, numerous studies provide evidence that combinatorial treatment of chemo-/radio-therapy or immunotherapy, together with phytochemical-mediated therapy, is beneficial and prolongs patients’ survival. Furthermore, the use of phytocompounds as photosensitizers provides a new way of addressing barriers in GBM therapy. Ultimately, the investigation of ‘forgotten compounds’ from lichens, mushrooms, or plants gives hope for a breakthrough in GBM therapy. In this article, we present the most up-to-date revision of the new, promising avenues and major achievements in phytocompounds research for GBM therapy, bringing the development of efficacious anti-GBM treatments closer.

## 2. Enhancing Temozolomide Response with Phytochemicals

TMZ is used as frontline chemotherapy in the management of GBM. However, 30–60% of GBM patients express O^6^-methylguanine-DNA methyltransferase (MGMT), a repair protein that reverses the alkylating changes introduced by the drug [11,12,13]. Thus, only a subset of patients is initially sensitive to TMZ treatment. Moreover, among the initially sensitive patients, TMZ resistance develops over time, creating a serious medical problem, taking into consideration the fact that a very limited number of other treatment options are available for GBM patients. The expression of multidrug resistance protein 1 (MDR1, ABCB1) creates an additional method of drug resistance and is responsible for the treatment failure [7]. Furthermore, glioma stem cells, a subset of undifferentiated cells that have the ability to both self-renew and produce clonal populations of differentiated tumor cells, are also well-known contributors to TMZ resistance [14,15]. TMZ resistance is a significant obstacle that must be overcome for the successful treatment of GBM [16]. Therefore, compounds able to enhance the TMZ response via downregulation of MGMT or ABCB1, and those able to reverse TMZ resistance or to target glioma stem cells, are intensively searched. A novel approach that has emerged recently aims to postpone the onset of acquired drug resistance, decrease the toxic side effects of anticancer medications, and boost the cytotoxic efficiency of these therapies by sensitizing cancer cells to conventional drugs through the use of non-toxic natural compounds [17,18,19,20]. Current data provide evidence that in the case of GBM cells, the improvement of the TMZ response and the reversal of drug resistance are possible using selected phytocompounds.

Recently, Gautam et al. found synergism between naringenin, a flavonoid found in grapefruit, and TMZ in restricting growth and enhancing cytotoxic effects against U87MG and LN229 cell lines [21]. Additionally, the study demonstrated a significant decrease in both single-cell colony formation and cell migration. In another study, Netto et al. evaluated the biological action of matteucinol (Mat), a flavanone found in *Miconia chamissois*, *Miconia trailii*, *Miconia prasina*, or *Rhododendron hainanense*, when used in combination with TMZ [22]. With the use of U-251MG human GBM cells, the authors discovered that the Mat–TMZ combination was cytotoxic and selective for tumor cells. Furthermore, the combination produced morphological alterations characteristic of apoptosis in vitro, but did not influence cell adhesion. Additionally, the combination also reduced tumor growth in the chicken embryo chorioallantoic membrane (CAM) assay [22].

Cordycepin is a major bioactive component in *Cordyceps militaris*, known for its immunomodulatory, anticancer, antioxidant, and anti-inflammatory properties [23]. Bi et al. investigated the molecular pathways underlying the anti-glioma effects of cordycepin and TMZ and assessed the effectiveness of their combined use in the treatment of GBM. According to the findings, cordycepin increases TMZ sensitivity in human glioma cells, at least in part, by suppressing the AKT signaling pathway and activating AMPK. Notably, the combination therapy reduced the tumor volume and prolonged the median survival time of GBM xenograft models [23]. In a different investigation, the capacity to decrease MGMT expression in glioma cells was assessed in regards to rutin, catechin, dehydrozingerone, naringenin, and quercetin treatment, both individually and in combination with TMZ [24]. Following a preliminary in silico screening of phytochemicals against MGMT, five compounds were selected on the basis of their favorable binding energies and high docking scores. Among them, quercetin was found to exert a synergistic effect with TMZ, increasing the cytotoxicity and inducing apoptosis of C6 cells. Moreover, quercetin, when combined with TMZ, significantly decreased the MGMT levels in C6 cells, as estimated by ELISA. The findings of this investigation suggested that using TMZ in combination with quercetin may mitigate TMZ resistance [24].

In another study, genistein and TMZ-loaded polymeric nanoparticles (Gen-TMZ-NPs) were evaluated for improved anti-tumor efficacy against GBM cells [25]. Gen-TMZ-NP treatment decreased cell proliferation, induced apoptosis, and reduced migration of U87MG cells [25]. In addition, research was carried out on cedrol, a sesquiterpene alcohol obtained from *Cedrus atlantica*, as a possible new agent for GBM therapy in combination with TMZ [26]. The evaluated TMZ–cedrol treatment reduced GBM cell proliferation via regulation of the AKT and MAPK signaling. Additionally, investigators noted that when both cedrol and TMZ were utilized, the effects of cell-cycle arrest, apoptosis induction, and DNA damage were more noticeable than when either compound was used alone. Moreover, in TMZ-treated GBM cells, cedrol decreased the expression of proteins linked to drug resistance, such as MGMT, MDR1, and CD133. In addition, the animal model demonstrated that the combined therapy greatly inhibited the growth of tumors [26].

Additional studies evaluating the efficacy of the combined therapeutic strategies utilizing TMZ or another anticancer drug, together with a phytocompound, are presented in Table 1. When phytocompounds were used in conjunction with chemotherapeutic drugs to treat cancer cells, the result was typically an increased cytotoxic effect. This was brought about by activating alternative signaling pathways that cause cell death or by prolonging the anticancer drug’s residence time in the cell, which enhanced its effectiveness.

## 3. Strategies for the Improved Phytocompound Delivery to the Brain—The Advantages of Phyto-Nanocarriers

One of the major challenges in GBM treatment is the presence of the BBB and the BTB. Drugs must also penetrate the tumor core after entering the brain in order to minimize harm to normal brain tissue and obtain effective therapeutic outcomes. In this regard, novel pharmaceutical approaches, such as the encapsulation of drugs in nanoformulations, have been proposed. In the context of cancer phytotherapy, this approach has an additional advantage, as it can overcome the intrinsic drawbacks of bioactive compounds, including low solubility, structural instability, short half-life, poor bioavailability, and poor targeting to specific organs [10]. Indeed, the enormous potential of phytocompound-encapsulated nanoformulations is evident from the publication trends over the last decade [10,50,51].

In a study by Piwowarczyk et al., four phytocompounds, namely curcumin, bisdemethoxycurcumin, acteoside, and orientin, as well as their mixtures, were encapsulated in a cationic liposomal nanoformulation and were evaluated on T98G and U-138 MG GBM cell lines [52]. After incubating for 24 h, liposome-entrapped acteoside showed the strongest anticancer activity against the T98G cell line, with an IC_50_ of 2.9 ± 0.9 µM. Furthermore, the combination of curcumin and orientin in the liposomal formulation had a synergistic effect on GBM cells. Additionally, acteoside and the mixture of curcumin and orientin were both found to induce apoptosis, as they increased the expression of p53 and caspase-3 [52]. In another study, Ying et al. used ursolic acids and EGCG encapsulated in liposomes to inhibit growth and induce apoptosis of C6 glioma stem cells [53]. Further experiments using animal models showed that this liposomal treatment inhibited tumor growth and increased the survival time of brain glioma-bearing mice [53]. Evidence also exists showing that curcumin-loaded niosome nanoparticles exerted significantly higher effects on glioma stem cells’ viability, apoptosis, and cell cycle, as compared to free curcumin [54]. Additionally, the migration and colony formation of glioma stem cells were significantly impaired following the administration of curcumin nanoparticles. These results clearly show that the cytotoxic effects of curcumin were markedly enhanced when loaded in niosome nanoparticles [54].

Instead of using single phytocompounds, or a combination of phytocompounds, another strategy in GBM research is to use combination therapies of anticancer drugs together with a phytocompound. Such a combination can be delivered to brain tumors using nanoformulations, which allows lowering the effective dosage of the anticancer drug and reducing the systemic drug-induced toxicity. In this regard, Ismail et al. tested a combined delivery of artesunate, a derivative of artemisinin, and TMZ using in vitro and in vivo GBM models [55]. The authors created the ApoE-functionalized liposomes based on artesunate-phosphatidylcholine encapsulated with TMZ. Crucially, those liposomes were successful in crossing the BBB by means of transcytosis mediated by low-density lipoprotein family receptors (LDLRs), which allowed for deep cerebral tumor penetration. Eventually, the authors showed that such a nanoplatform can successfully co-deliver dual therapeutic agents to TMZ-resistant U251-TR GBM cells in vivo [55].

In another study, a combined treatment of curcuminoids and sodium butyrate was evaluated on human GBM cells and revealed that sodium butyrate ameliorated curcuminoids’ permeability through the BBB and the nasal cavity [56]. The data provided evidence that curcuminoids and sodium butyrate work together to decrease GBM cells’ viability by causing apoptosis and cell-cycle arrest. According to the study’s findings, ROS generation and alterations in gene expression, such as the overexpression of *SFRP1*, *RUNX3*, and Wnt/β-catenin pathway antagonists, and the downregulation of *UHRF1*, the primary epigenetic regulator, mediate these effects [56].

Currently, targeting or cell-penetrating molecules, such as antibodies, antigen-binding fragments (Fab’), peptides, and small molecules, are used to construct targeted liposomes that actively bind to or penetrate target tissues or cells. In a study by Liu et al., a specific ligand cyclic RGD peptide was conjugated to a cell-penetrating peptide, R8, to develop a multifunctional peptide, R8-RGD [57]. This tandem peptide conjugation increased the cellular uptake of liposomes by 2-fold and nearly 30-fold compared to separate R8 and RGD, respectively. It also displayed effective penetration of three-dimensional glioma spheroids and a BBB model in vitro [57]. In another study, resveratrol was incorporated into the lipid bilayer of epirubicin-encapsulating liposomes [58]. The liposomes were further modified with p-aminophenyl-α-D-manno-pyranoside (MAN) and wheat germ agglutinin (WGA) on their surface. The authors provided evidence that the epirubicin plus resveratrol liposomes modified with WGA and MAN exhibited a strong ability to improve epirubicin and resveratrol transport across the BBB. Importantly, the experiments with C6 glioma-bearing rats showed that the survival time of rats treated with the tested liposomes was longer than that of control groups [58]. Even though, currently, there are no phytocompound-based nanoformulations approved by the FDA to treat brain tumors, the examples from other cancer types are encouraging. Liposomal vincristine for acute lymphoid leukemia (Marqibo^®^), and paclitaxel-bound albumin (Abraxane^®^) and paclitaxel-loaded polyethylene glycol-polylactic acid (PEG-PLA) polymeric micelle (Genexol-PM^®^) for breast and lung cancer, are already used in clinics, instilling optimism that the phytocompound-based nanoformulations will soon also be available for GBM tumor patients.

## 4. Chemical Modifications of Phytocompounds as a Way to Improve Their Solubility, Bioavailability, and Efficacy

Phytocompounds target multiple signaling pathways deregulated in cancer cells, demonstrating, in the vast majority of cases, selective toxicity toward cancer cells. In this regard, ERK1/2 signaling, the Wnt/β-catenin pathway, and the PI3K/AKT pathway are among the signaling pathways relevant to GBM that can be targeted by the phytochemicals. However, the poor solubility and low bioavailability of phytocompounds represents a major disadvantage for their clinical application. Thus, chemical derivatization of natural compounds is a promising strategy to improve the solubility, bioavailability, and target specificity and, eventually, to obtain more effective anticancer drug candidates.

In this regard, five synthetic analogs of resveratrol, i.e., 3,4,4′-trimethoxy-stilbene, 3,4,2′-trimethoxy-stilbene, 3,4,2′,4′-tetramethoxy-stilbene, 3,4,2′,6′-tetramethoxy-stilbene, and 3,4,2′,4′,6′-pentamethoxy-stilbene, were studied using human GBM T98G cells [59]. The results of this study revealed that methoxy-stilbenes are characterized by high BBB permeability and influence the Wnt/β-catenin pathway in GBM cells. The 3,4,4′-trimethoxy-stilbene was the most effective inhibitor of the Wnt/β-catenin pathway among all the chemicals examined. Crucially, administration of 3,4,4′-trimethoxy-stilbene and 3,4,2′,4′-tetramethoxy-stilbene resulted in apoptosis and cycle arrest in the S phase. While all the chemicals caused moderate alterations in the level of expression of the epigenetic modifiers *DNMT3B* and *TET1-3*, none of them had an effect on the DNA methylation level of *MGMT*, *SFRP1*, or *RUNX3* [59].

Rampogou et al. have synthesized three analogs of butein—a flavonoid known to have anticancer properties, exhibited by apoptosis induction, COX-2 repression, and reactive oxygen species (ROS) production, among others [60]. Using the Discovery Studio, butein has been modified at ring B position to obtain new derivatives. These three compounds were then evaluated for their anticancer properties targeting RNA helicase, DDX3. This protein is overexpressed in several cancers, including GBM, and is regarded as a validated target to design and develop anticancer drugs [61,62]. It has been found that in GBM cells, DDX3 induces epithelial–mesenchymal transition (EMT) through the DDX3/snail/E-cadherin axis [61]. Using the CDOCKER program, Rampogou and coauthors elucidated the binding affinity of the three novel butein analogs toward DDX3. Additional analysis revealed that these compounds had arrested the cell cycle and induced apoptosis in MCF-7 and MDA-MB-231 cell lines. Eventually, two butein derivatives were selected as novel DDX3 inhibitors [60]. Whether these two compounds exhibit inhibitory activity toward DDX3 in GBM cells awaits elucidation, but the results obtained in a breast cancer model are promising.

Nowadays, bioinformatics represents a valuable approach to facilitate and speed up the identification of novel derivatives of natural compounds. Utilizing an array of advanced computational methodologies, such as machine learning algorithms and data analytics, it is possible to carry out the analysis of hundreds of compounds, selecting the most potent ones for further in vitro and in vivo analysis [63]. Such an approach facilitates the research of targeted phytocompound-based drugs to treat GBM.

Following this approach, 50 flavonoids and polyphenol derivatives, mostly from plant sources, were subjected to target-based virtual screening [64]. The study examined how these substances interacted with protein tyrosine phosphatase receptor-type Z (PTPRZ) using molecular docking and molecular dynamic modeling approaches. The authors utilized sophisticated computational tools, specifically PyRx and the AutoDock Vina wizard, to achieve a more detailed understanding and guarantee the accuracy of their findings. The outcomes highlighted luteolin and ferulic acid’s coordinated inhibitory dynamics, particularly with relation to their binding energies during drug–target interactions [64].

In this regard, the in silico drug screening of fifty curcumin derivatives was performed in order to identify compounds with better binding affinities to EGFR and NF-κB [63]. Subsequently, the in silico results were exemplarily validated using microscale thermophoresis. Next, the bioactivity was examined using the annexin V/propidium iodide assay, lactate dehydrogenase assay, resazurin cell viability assay, and flow cytometric measurement of reactive oxygen species. The study revealed, among other things, that whereas distinct curcumin compounds have the same upstream targets (EGFR and NF-κB), they may activate distinct downstream pathways. Remarkably, N-(3-nitrophenylpyrazole) curcumin, one of the derivatives, produced a non-apoptotic, ROS-independent route of cell death. This discovery might have significant therapeutic benefits. The authors concluded that molecular docking is a useful method for facilitating and accelerating the discovery of new, targeted, curcumin-based cancer treatment medications.

Caffeic acid phenethyl ester (CAPE), a natural bioactive compound found in propolis, penetrates the BBB and has a broad anticancer effect on various cancer cell lines [65]. For instance, in a study by Lee et al., it was shown that CAPE induces p53-dependent apoptosis in C6 glioma cells, influencing the p38 MAPK signaling [66]. However, CAPE has crucial solubility issues; therefore, new CAPE analogs with increased solubility are needed. In this regard, Sucu et al. synthesized several novel CAPE analogs and evaluated their therapeutic efficacies in GBM cell lines (T98G and LN229). Compound 10 was found to be the most potent in the context of viability reduction and apoptosis induction. Further molecular docking and molecular dynamics simulation studies on NF-κB, EGFR, TNF-α, ERK2, PAPR1, hCA IX, and hCA XII targets playing roles in GBM revealed that CAPE analogs are able to interact with critical residues in binding pockets of the above-mentioned targets. The authors concluded that CAPE analogs, in particular heterocyclic analogs, are promising anti-GBM agents and deserve further investigation [65].

## 5. Phytocompound-Based Immunotherapy

Even though the traditional therapies, such as surgery, chemotherapy, and radiation, remain the first-line approaches, nowadays, new immunotherapy strategies are considered very promising in the treatment of GBM [67]. Immunotherapy strategies, such as immune checkpoint inhibitors (ICIs), vaccines, chimeric antigen receptor T-cell (CAR-T cell) therapy, and virus therapy, are already being tested in clinical trials. In the meantime, the role of natural products in glioma immunotherapy is being intensively studied. Phytocompounds possess immunoregulatory properties by reversing the glioma immunosuppressive microenvironment (GIME), composed of myeloid-derived suppressor cells (MDSCs), M2-type tumor-associated microglia/macrophages (TAMs), and Tregs [68]. The following mechanisms have been described regarding the role of phytocompounds in regulation of the immune system: (i) remodeling TAMs, (ii) inhibiting myeloid-derived suppressor cells (MDSCs) and Tregs, (iii) reactivating immune effector cells (including T cells and natural killer cells (NK cells)), and (iv) modulating immune-related signaling pathways in glioma cells [68].

One of the compounds intensively studied in this context is chlorogenic acid, a phenolic compound found in many plants. As a potential cancer immunotherapeutic agent, chlorogenic acid has entered phase II clinical trial in China as a lyophilized powder formulation for treating glioma [69]. However, the in vivo instability of chlorogenic acid necessitates daily intramuscular injections, resulting in patient noncompliance. That is why novel formulations are being developed that could reduce the frequency of drug administration. One of them was recently developed by Zhang et al., who prepared chlorogenic acid-phospholipid complex-containing PEGylated liposomes (CPPL) [69]. CPPL demonstrated excellent physicochemical properties, enhanced tumor accumulation, and inhibited tumor growth, even when the administration interval was prolonged to four days. The molecular mechanism responsible for the immunotherapeutic effect included stimulating both CD4+ and CD8+ T cell infiltration, inhibiting MDSCs’ expression, reducing the expression of Th2-related factors, and notably, increasing the memory T cells in tumor tissues [69].

Creating mannosylated liposomes to deliver chlorogenic acid to TAMs was yet another proposal [70]. The research demonstrated that mannosylated liposomes encapsulated in chlorogenic acid effectively promoted the polarization of the pro-tumorigenic M2 phenotype toward the anti-tumorigenic M1 phenotype, thereby inhibiting the growth of G422 glioma tumors. Ultimately, the study showed that mannosylated liposomes encapsulated in chlorogenic acid have a tremendous potential to increase the immunotherapeutic efficacy of chlorogenic acid by causing a shift in phenotype from M2 to M1 [70].

Mukherjee et al. synthesized liposomes encapsulating curcumin, resveratrol, and epicatechin gallate (TriCurin) to examine its anti-tumor efficaciousness on GBM cells both in vitro and in vivo [71]. It was found that TrCurin could induce activated NK cells to be recruited into the tumor, and the repolarization of pro-tumor M2-like TAMs to the tumoricidal M1-like phenotype was also observed [71]. Such tumoricidal immune cells have been shown to concurrently decrease tumor load and induce GBM and GBM stem cell apoptosis when they are present intratumorally. The authors concluded that TriCurin is a potential onco-immunotherapeutic agent against GBM tumors [71].

In another study, apigenin, the flavonoid from the Brazilian plant Croton betulaster Müll., was evaluated in the context of chemotaxis and regulation of inflammatory cytokines of microglia cells [72]. The study demonstrated that apigenin administration decreased the expression of CD206 (an M2 profile marker) on microglia and raised the expression of OX-42 and iNOS (M1 phenotypic markers), suggesting that apigenin plays a function in enhancing the microglia-activated phenotype. Additionally, the scientists presented proof that apigenin-treated microglial cells are activated and possess chemotaxis toward glioma cells. Additionally, they discovered that apigenin enhanced microglia differentiation and reduced glioma cell viability, which was connected with the ratio of TNF and IL-10 produced by microglia [72].

Most recently, Yi et al. evaluated a novel anti-GBM strategy based on a combined administration of interferon-elastin-like polypeptide (IFN-ELP(V)) and resveratrol. IFN-ELP(V) is a slow-release, biodegradable, thermosensitive fusion protein designed for intratumor injection, whereas resveratrol was administered as an intraperitoneal injection [73]. The results of this study suggested that IFN-ELP(V) can create a reservoir in the tumor, in which IFN is continuously released to produce a powerful in situ anti-tumor immune response, whereas resveratrol synergistically augments the anti-tumor effect. Using a mouse model, the authors showed that the intratumor injection of IFN-ELP(V) combined with the intraperitoneal injection of resveratrol was effective in delaying GBM growth. Such an approach provides a novel and effective therapeutic strategy for tumors, especially those that are unsuitable for surgical resection [73].

## 6. Phytocompounds Used as Photosensitizers in GBM Photodynamic Therapy (PDT)

Various natural compounds exhibit photosensitizing potentials and can be used in photodynamic therapy (PDT) [74]. As compared to synthetic photosensitizers, phytocompounds are regarded as safer and generate fewer side effects. Furanocoumarins, polyacetylenes, thiophenes, curcumins, alkaloids, and anthraquinones possess both antineoplastic and phototoxic properties and have been tested as potent photosensitizers for an effective PDT outcome in the treatment of various cancers [75,76]. The photoactivity of natural products is only recently shifting back into focus as a treatment option against GBM. It is an especially promising approach in the context of elimination of the remaining infiltrative so-called guerilla cells of the tumor margin, which due to diffuse growth of the tumor cannot be removed during surgical resection. Thus, we observed a trend toward continuation of the efforts to apply PDT in the treatment of GBM, and phytocompounds such as curcumin and hypericin are evaluated as potent photosensitizers. In this regard, in a study by Kielbik et al., the application of a photodynamic reaction with curcumin as a photosensitizer significantly decreased the cancer cell viability after 2 h of incubation compared with the non-irradiated group [77]. The photosensitization enabled the use of a 6.3 times lower concentration of the drug to decrease the tumor cells’ viability by 50% compared with curcumin therapy without radiation [77].

Moreover, an older study, in which an alkaloid berberine was analyzed, revealed that berberine successfully accumulates in the mitochondria of the human primary GBM U87MG cell line and, upon irradiation, shows phototoxicity, leading to apoptosis [78]. Newer studies on the use of berberine in PDT of gliomas, however, are scarce.

Shikonin, one of the main active ingredients of Chinese herbal medicine *Lithospermum erythrorhizon*, was also evaluated in the context of PDT of GBM [79]. In a study by Werner et al., in vitro experiments using U87 MG and the primary glioblastoma cell line GB14 were conducted to examine the effects of shikonin on cancer cells’ viability, proliferation, induction of apoptosis, and tumor sphere formation capacity. The impact of the combination of PDT and TMZ on the mRNA expression of stem cell markers specific to gliomas was also studied. The study revealed that shikonin improved 5-aminolevulinic acid-based PDT via modulation of the intracellular protoporphyrin IX levels. It also effectively inhibited the capability of forming tumor spheres and enhanced TMZ’s effectiveness via the reduction of proliferation and induction of apoptosis [79].

On the other hand, hypericin is regarded as an excellent photosensitizer and undergoes intensive evaluation in the context of PDT of GBM. Recently, the mechanism of hypericin-induced PDT was investigated in single glioma cells [80]. A sequence of steps that cause cell damage was revealed by the spatially resolved fluorescence lifetime and spectroscopic examination of hypericin-induced PDT on glioma cells. These steps include membrane bleb formation, ER degradation, cell shrinkage and rounding, and breakdown of membrane stability, which leads to flooding of the cell with the surrounding medium [80]. Therefore, it was discovered that apoptosis followed by necrosis were the main causes of cell death in hypericin-induced PDT on glioma cells [80].

It has been reported that photo-biomodulation (PBM) with light at 808 nm has beneficial effects on damaged mitochondria in cells [81]. PBM has grown in popularity as a technique for controlling reactive oxygen species in cells in recent years. In this context, Pevna et al. combined hypericin-mediated PDT (hypericin-PDT, 2 J/cm^2^) of U87MG cells with (2 min, 15 mW/cm^2^ at 808 nm) photo-biomodulation (PBM) [81]. They found that PBM induces autophagy, which when combined with PDT boosts therapy effectiveness and causes GBM cells to undergo apoptosis [81].

The comprehensive understanding of the photosensitizer–tumor interactions is necessary for successful PDT using phytocompounds. In order to better understand the effects of hypericin PDT for glioma tumors, Bassler et al. performed a study concerning the accumulation and penetration behavior of hypericin in GBM cells using an improved in vitro spheroid tumor model [82]. They found that low incubation concentrations and short incubation times caused an increased enrichment at the peripheral region of the spheroids, with hypericin gradients toward the center. In contrast, high incubation concentrations and long incubation periods led to a more homogeneous hypericin distribution throughout the spheroid.

For additional information about natural and synthetic photosensitizers in the treatment of GBM, we recommend the review article in [83].

## 7. Unraveling the Emerging Role of Less Studied Plant-Derived Substances with Anti-GBM Potential

Despite many new therapeutic concepts, GBM remains one of the most difficult cancers to treat. Thus, the search for new natural active substances with anti-GBM potential is still a valuable research direction. It allows to select further previously unknown molecules, which can be further tested in the search for more effective anti-GBM therapies.

In 2019, Afshari et al. published a paper describing, for the first time, the effect of auraptene on GBM cell lines [84]. This coumarin (geranyloxycoumarin), found in plants belonging to *Rutaceae* and *Apiaceae* families, reduced the viability of U87 cells in a concentration- and time-dependent manner. Moreover, after treatment with the above-mentioned compound, the cells were arrested in the sub-G1 cell-cycle phase. Upregulation of *Bax*, *NF-κB*, and *IL-1β*, and downregulation in *MCP-1* and *Bcl-2* expression, were also observed [84]. Additionally, in a recently published article, Izadi et al. proved that the combination therapy using alpha-lipoic acid and auraptene reduced MMP-2/-9 expression and their enzymatic activity, effectively inhibiting the migration and metastasis of U87 cells [85].

In another article, scientists from Greece studied the leaves and flowers of the perennial plant *Achillea grandifolia* [86]. Isolation work allowed them to isolate several compounds, of which rupicolin A and rupicolin B, two main secondary metabolites belonging to the guaianolides group, were tested on U87MG and T98G glioblastoma cell lines. The IC_50_ values calculated based on the MTT test were 38 μM and 64 μM for the U87MG cells, and 15 μM and 26 μM for the T98G cells, respectively. In addition, both compounds tested induced a G2/M cell-cycle arrest [86].

Sixteen diterpenoids isolated from the aboveground parts of *Caesalpinia pulcherrima* (L.) were studied by Chen et al. [87]. One of the new compounds, pulcherritam A, as well as pulcherrimin G, previously isolated from this species, showed interesting anti-GBM activity, with IC_50_ values of 10.5 ± 0.23 μM and 10.7 ± 0.28 μM, respectively, established for the U87MG cell line [87].

One of the more recent studies assessed the anti-glioma potential of eucalyptal A, a compound with phloroglucinol-terpene skeleton isolated from the fruit of *Eucalyptus globulus*. Hua et al. tested its anti-GBM activity using in vitro LN229 and U87MG cell lines and in vivo mouse models. The results demonstrated the inhibitory impact on the proliferation, growth, and invasiveness of GBM cells. The authors revealed that eucalyptal A downregulated serine/arginine-rich splicing factor 1 (SRSF1) expression and rectified SRSF1-guided abnormal alternative splicing of MYO1B mRNA, which led to anti-GBM activity through the PDK1/AKT/c-Myc and PAK/Cofilin axes. Importantly, eucalyptal A exhibited stronger anti-GBM activity and lower toxicity, as compared to TMZ [88].

Still, little is known about galangin, 3,5,7-trihydroxyflavone, which is a flavonoid present in *Alpinia officinarum*, one of the Asian therapeutic plants. Chen et al. proved that galangin has anti-GBM potential tested in U87 and U251 cell lines, and the mechanisms responsible for this effect include inhibition of proliferation, migration, invasion, and angiogenesis of GBM cells. The results also showed that galangin is able to downregulate the expression of CD44 and EMT markers in GBM cells. Importantly, the in vivo study, using an orthotopic xenograft mouse model, indicated that galangin suppresses tumor growth and increases the survival of GBM-bearing mice [89]. Other independently performed studies confirmed the ability of galangin to reduce the viability and proliferation of GBM U251, U87MG, and A172 cells (without affecting normal human astrocytes), as well as confirmed its ability to induce apoptosis and pyroptosis of GBM cells. Moreover, using an orthotopic xenograft mice model, the authors showed that galangin was effective in decreasing tumor growth when applied with an inhibitor of autophagy, chloroquine [90]. Further studies also identified Skp2, a pivotal component of SCF^Skp2^ E3 ubiquitin ligase, as a novel target of galangin for the treatment of GBM [91]. Results from this study indicated the potential of galangin for the treatment of GBM through ubiquitin-mediated degradation of Skp2 [91].

In another recent study, casticin, a methoxylated flavonol compound isolated from the fruits of a traditional Chinese medicine *Vitex rotundifolia* L., was tested using U87MG, U251MG, and LN229 GBM cell lines. The results indicated that casticin inhibited tumor cell growth in vitro and in vivo. Moreover, apoptosis and autophagy induction were shown after treatment with casticin. The results obtained by Yang et al. also showed that casticin inhibited the Akt/mTOR and JAK2/STAT3 signaling pathways and reduced the glioma stem cells population by suppressing Oct4, Nanog, and Sox2. Hence, it may arouse interest as a new anti-GBM agent [92].

Atranorin is a common lichen secondary metabolite [93]. However, the literature data proved the presence of this compound in a few plant species [94,95]. The anti-GBM activity of this compound was presented by Majchrzak-Celińska et al. [96]. A-172, T98G, and U-138 MG cell lines were found to be cytotoxically susceptible to atranorin (IC_50_ = 90.89 ± 3.70, 98.63 ± 7.80, and 47.84 ± 2.16 μM, respectively). Additionally, atranorin changed the T98G cell line’s distribution of cell-cycle phases and improved GBM cells’ response to TMZ treatment, and these results were mediated via the Wnt pathway inhibition [96]. In addition, lichens, symbiotic organisms composed of fungal and algal (e.g., green algae) cells, produce depsidones (physodic acid and salazinic acid), depsides (evernic acid, lecanoric acid, and squamatic acid), dibenzofuran derivatives (usnic acid), and fatty acids (caperatic acid), which decrease GBM cell viability to varying degrees [97,98,99], and some of them also disrupt the cell cycle, induce apoptosis, or inhibit the Wnt/β-catenin pathway, enhancing the effects of TMZ [96].

In addition to testing pure compounds of plant origin, the activity of plant-derived extracts is also being evaluated. Extracts constitute an interesting, natural matrix containing many different compounds. Therefore, the effect of the extract often differs from the action of pure compounds and often refers to the synergistic effect of individual substances.

A recent study performed by Murugesan et al. reported that a polyphenol-rich fraction obtained using alcoholic extraction from one of the marine halophytes, *Ipomoea pes-caprae*, is a promising source of compounds with anti-glioma potential. Experiments conducted on two human glioma cell lines, LN229 and SNB19, showed that the *Ipomoea pes-caprae* extract exhibited anti-proliferative and anti-metastatic properties in GBM cells [100]. The study revealed that the extract induces phosphorylation of MMK3, p53, p70 S6 kinase, and RSK1 proteins, modulates the MAP kinase signaling pathway in GBM cells, and induces cell death via the ROS-mediated, caspase-dependent pathway [100].

Interesting results were also provided by Kakouri et al. [101]. For the first time, they described the anti-GBM activity of a 70% methanol extract from the aerial part (leaves and stems) of *Smilax aspera*, which is rich in flavonoids, especially in rutin and luteolin. The A172 cell line was selected for the biological study, and the detected IC_50_ after 48 h of treatment was 0.482 ± 0.98 mg/mL [101].

Another group of compounds with high anti-GBM activity is gallotanins, for example, found in *Quercus infectoria* galls [102,103]. Kamarudin et al. obtained the fractioned extract rich in gallotanins and showed its high cytotoxicity (IC_50_ 15.0 μg/mL) in the in vitro tests using the DBTRG-05MG cell line. Indeed, the fractionated extract was found to significantly suppress GBM cell growth, better than the synthetic pure gallotannin and the *Quercus infectoria* gall crude extract. Moreover, the inhibitory effects exerted by the fractionated extract treatment on GBM cells were comparable to the effects of TMZ [102].

## 8. Epidemiological Studies of Dietary Phytocompounds—Novel Trend in GBM Research?

The epidemiological studies on dietary phytochemicals intake in GBM patients are scarce. However, most recently, an interesting study was published by Zhang et al., presenting the results from dietary mixed exposure to phytochemicals among glioma patients [104]. The researchers estimated the phytochemical intake of 506 patients with glioma and 506 controls. The findings demonstrated that for GBM, carotene, flavonoids, soy isoflavones, and anthocyanins were related to a significantly reduced risk. For astrocytoma, resveratrol was also significantly related to a reduced risk. The examination of the dose–response relationship showed that the risk of glioma tended to decrease when the daily consumption of carotenes exceeded 1186.16 μg. Similar to carotene, when the consumption of flavonoids exceeded 52.48 mg/d, the incidence of gliomas decreased. As far as soy isoflavones are concerned, the risk increased at first and then decreased, and this downward trend gradually weakened when the intake exceeded 6.14 mg/d. For anthocyanins, when the intake exceeded 4.53 mg/d, the risk tended to decrease as intake increased. Similar to anthocyanins, resveratrol also exhibited a notable L-shaped trend. When intake exceeded 572.39 μg/d, the risk tended to decrease as intake increased. Following an intake above 1085.51 μg/d, the risk of glioma remained very steady [104]. Future prospective studies should further confirm these relationships.

## 9. Phytocompounds or Phytocompound-Based Products That Reached the Clinical Trials Phase in GBM Research

Among the hundreds of plants evaluated in cancer research, only a small number pass the laboratory experiments, reaching the phase of clinical trials [105]. Nevertheless, as presented by Hosseini and Ghorbani, there is clinical evidence supporting the anticancer effects of camptothecin, curcumin, resveratrol, *Allium sativum*, *Panax ginseng*, *Rhus verniciflua*, *Viscum album*, and green tea [105]. However, clinical trials evaluating the role of phytocompounds or medicinal plants with anti-GBM properties are scarce (Table 2). In 2006, Guzmán et al. reported the first clinical study aimed at assessing cannabinoid, i.e., Δ9-tetrahydrocannabinol (Δ9-THC), anti-tumoral action in patients with recurrent GBM [106]. In this pilot clinical study, Δ9-THC was administered intratumorally to nine individuals with recurrent GBM. The patients had previously failed standard therapy, which included radiation and surgery, and clearly showed signs of tumor growth. The safety of intracranial Δ9-THC administration was the study’s main goal, while other end points included tumor cell parameters and patients’ survival time analysis. The clinical study’s findings showed that Δ9-THC delivery was safe and achievable without producing overtly psychoactive side effects, with the cohort’s median survival from the start of THC treatment of 24 weeks (95% confidence interval: 15–33). Moreover, Δ9-THC suppressed tumor cell growth in vitro and decreased tumor cell Ki67 immunostaining when administered to two patients [106].

Not only Δ9-THC, but also CBD has been evaluated in clinical trials in the context of GBM treatment. The molecular mechanisms responsible for the anti-tumor effect exerted by CBD include, among others, targeting glioma stem cells and cancer-related signaling pathways [107,108], influencing the tumor microenvironment (TME) [109], and producing reactive oxygen species (ROS) in tumor cells [110]. Overall, numerous in vitro and in vivo studies showing beneficial effects of CBD against GBM cells (just to mention a few most recent studies [107,109,110,111,112]) paved the way for clinical trials evaluating the impact of this cannabinoid on humans.

In September 2023, an 8-week, double-blind, placebo-controlled, randomized clinical trial (ClinicalTrials.gov Identifier: NCT05753007) was initiated to evaluate the effect of a custom formulated, full-spectrum, hemp-derived, ultra-high CBD product on anxiety, pain, and quality of life in newly diagnosed GBM patients receiving standard-of-care treatment. It will also be evaluated how this product affects the growth of tumors in comparison to a placebo. This clinical trial will offer crucial data on the possible effectiveness of a unique full-spectrum, ultra-high CBD medication to treat clinical symptoms in GBM patients.

Additionally, a randomized, double-blind, placebo-controlled, parallel, multi-center study (ClinicalTrials.gov Identifier: NCT03607643) was registered in 2018 to assess the efficacy of CBD (BRCX014) combined with standard-of-care treatment in subjects with GBM, multiple myeloma, and gastrointestinal malignancies; however, no results of this study are available yet.

Following its approval for sale in the US in June 2018 and the EU in September 2019, phyto-CBD is now freely accessible for medical prescription in a number of nations, including Austria, Germany, and Switzerland [113]. Likar et al. retrospectively assessed the survival of a cohort of 15 consecutive, unselected patients with histopathologically confirmed GBM who received CBD (400 to 600 mg orally per day) in addition to standard therapy (maximum resection of the tumor, followed by radio-chemotherapy) [113]. Of the 15 patients, 7 (46.7%) had been alive for at least 24 months, and 4 (26.7%) had been alive for at least 36 months at the time of data collection. This was over two times longer than what had previously been documented in the literature [113]. More information about the application of CBD in GBM treatment can be found in a review article by Rybarczyk et al. [114].

Besides the use of either CBD or THC in addition to standard-of-care GBM treatment, the combination of both the above-mentioned phytocannabinoids is also being evaluated in clinical trials. A phase Ib, open-label, multicenter, intra-patient, dose-escalation clinical trial (ClinicalTrials.gov Identifier: NCT03529448) is currently being developed by the Spanish Group for Neuro-oncology (GEINO) to evaluate the safety profile of the THC + CBD combination at a 1:1 ratio, in addition to TMZ and radiotherapy, in patients with newly diagnosed GBM. The study’s rationale is that, in preclinical glioma models, THC and CBD have demonstrated an evident synergistic anti-tumor effect when combined with TMZ and radiation therapy. By binding to and activating the type 1 and 2 cannabinoid receptors (CB1 expressed in specific brain neuronal regions, and CB2 expressed in the immune system and in glial cells), THC and CBD have a wide range of biological effects. The activation of these receptors initiates a signaling pathway, called the endoplasmic reticulum stress response, which stimulates tumor cell autophagy. TRB3, a pseudo-kinase that has been shown to affect a number of signaling pathways, including ER stress, insulin resistance, autophagy, inflammation, and apoptosis, is responsible for mediating those effects [115]. The study is now recruiting patients, and the estimated completion of the study is December 2025.

In addition, the tolerability of cannabis with concurrent chemoradiation in the treatment of GBM was also assessed in the clinical trial (ClinicalTrials.gov Identifier: NCT03246113). The National Institute of Drug Abuse (NI-DA) provided a strain of cannabis for testing in this study that had a low THC content (compared to typical street cannabis) and a high CBD concentration. First, patients completed a cannabis sampling session to assess for initial marijuana tolerability. Proceeding this, patients completed 3–5 outpatient smoking sessions per week over a 6-week period. During each session, patients were allowed 90 min to smoke 0.5 to 2 cannabis cigarettes. Measures of pain, mood, nausea, quality of life, and the subjective effects of cannabis—both positive and negative—were among the outcome variables. The study was terminated in 2019; however, no results have been posted yet.

THC, CBD, and other cannabinoid and non-cannabinoid ingredients are present in the complex botanical preparation called Sativex^®^ ((Nabiximols oromucosal spray) GW Research Ltd., Cambridge, United Kingdom). Clinical trials evaluated the safety and preliminary efficacy of Nabiximols plus dose-intense temozolomide (DIT) in patients with first recurrence of GBM [116]. Part 1 of the study (ClinicalTrials.gov Identifier: NCT01812603) was open label, and Part 2 (ClinicalTrials.gov Identifier: NCT01812616) was randomized, double-blind, and placebo-controlled. Both required individualized dose escalation. Patients received Nabiximols (Part 1, n = 6; Part 2, n = 12) or placebo (Part 2 only, n = 9), with a maximum of 12 sprays/day with DIT for up to 12 months. Safety, efficacy, and TMZ pharmacokinetics (PK) were monitored. The study’s findings showed that the most common treatment-emergent adverse events were vomiting, dizziness, fatigue, nausea, and headache. In Part 2, 33% of both Nabiximols- and placebo-treated patients were progression-free at 6 months. Survival at 1 year was 83% for Nabiximols-treated and 44% for placebo-treated patients (*p* = 0.042), although two patients died within the first 40 days of enrolment in the placebo arm. There were no apparent effects of Nabiximols on the pharmacokinetics of TMZ. The promising results paved the way for another study (ClinicalTrials.gov Identifier: NCT05629702), which is now ongoing and recruiting patients. The trial, called ARISTOCRAT, is a phase II, multi-center, double-blind, placebo-controlled, randomized trial, aiming to compare the Nabiximols with placebo in patients with recurrent MGMT-methylated GBM, treated with TMZ. A target number of 234 patients will be randomly assigned, at a 2:1 ratio, to receive regular TMZ in addition to either Nabiximols or a Nabiximols-matched placebo. The study is expected to be finished in February 2027.

Besides cannabinoids, chlorogenic acid also reached the clinical trial phase. The goal of a study launched in 2016 was to ascertain a chlorogenic acid injection dosing regimen, to verify the maximum tolerated dose (MTD) and dose-limiting toxicity (DLT), as well as to provide pharmacokinetic characteristics in advanced GBM patients. The study is completed; however, the results have not been published yet.

Another natural compound that reached the clinical trial phase is curcumin. A study completed in 2013 (ClinicalTrials.gov Identifier: NCT01712542) measured the bioavailability of orally administered curcumin in the tumors of GBM patients. Currently, the tolerability, safety, and efficacy of liposomal curcumin in combination with radiotherapy and TMZ in patients with newly diagnosed, high-grade gliomas are being evaluated (ClinicalTrials.gov Identifier: NCT05768919). The study is now recruiting patients, and the estimated study completion date is May 2027.

## 10. Conclusions

The gold standard GBM treatment regimen only modestly prolongs patients’ overall survival, so there is a need for adjuvant therapies. Phytocompounds, exerting pleiotropic effects and targeting multiple cancer-related signaling pathways, represent a valuable source of novel anti-GBM therapeutics. Thus, a tremendous number of research groups have evaluated the molecular mechanisms responsible for their anticancer effects. In this regard, growing evidence demonstrates that cytostatic effects of phytocompounds are based on targeting glioma stem cells and modulating oxidative stress, inflammation, autophagy, and apoptosis, among others. Additionally, some phytochemicals are derivatized and undergo structural modification for increased potency. These innovative phytocompound-based chemical compounds can effectively address issues, such as inadequate brain bioavailability due to poor absorption and instability at physiological pH, high rates of metabolism, rapid systemic elimination, and restricted BBB permeability. These days, cheminformatics and bioinformatics can expedite and streamline the exceedingly intricate, costly, and time-consuming drug development processes. Developing novel derivatives with enhanced binding capabilities to proteins related to cancer is thought to be the first step toward creating phytocompound-based medications with better pharmacological characteristics. Additionally, new drug carriers are being developed to increase the deposition of phytocompounds in the tumor niche. With the use of nanoformulations, it is possible to improve the bioavailability and enhance the anti-tumor activity of phytocompounds. Additionally, chemo–herbal combination therapies are being intensively evaluated, showing promising results. It has been shown that such combinatorial treatment of chemotherapy, as well as radio- or immuno-therapy, together with phytochemical-mediated therapy, are beneficial and prolong patients’ survival. Furthermore, among the novel trends, we also observed the use of phytocompounds as immunomodulatory agents or as photosensitizers in PDT. Deciphering the role of less studied compounds also opens new avenues in anti-GBM drug discovery and development.

However, much remains to be learned about the pharmacokinetics, drug interactions, ideal dosages, long-term safety, and adverse effects of phytochemicals proposed for GBM treatment. Upcoming clinical trials on this topic are particularly warranted. Moreover, reversing GBM-related epigenetic dysfunctions using phytocompounds emerges as a promising method to overcome drug resistance. However, still more well-designed in vitro and in vivo studies are needed to fully decipher the role of the most promising compounds and their formulations in GBM treatment. Eventually, more well-designed clinical trials are necessary to apply plant-derived compounds into drug translation.

## Figures and Tables

**Table 1:** Combined therapeutic strategies evaluated within the last six years in preclinical studies of GBM treatment.

Compound	Group	Cell Line	Model	Results	Reference
Bevacizumab + elagic acid	phenolic acid	C6	in vitro	Antiproliferative efficacy. Inhibition of MGMT expression and time-dependent inhibition of MDR1.	[27]
5-Fluorouracil + thymoquinone	quinones	U-251MG	in vitro	Reduced cell viability and proliferation in GBM cells. Strong synergistic anticancer effect.	[28]
Irinotekan + elagic acid	phenolic acid	C6	in vitro	Synergistic effect. Reduced cell proliferation by inhibiting the cadherin switch and promoting the antiangiogenic processes.	[29]
Temozolomide + berberine	alkalod	U87 U251	in vitro/ in vivo mice	Enhanced autophagy and apoptosis in TMZ-resistant cells linked with ERK1/2 signaling. In vivo increased GBM sensitivity to TMZ through the ERK1/2 signaling pathway.	[30]
Temozolomide + harmine	alkaloid	T98G	in vitro	Decreased cancer cells’ migration, invasion, and adhesion potentials, as well as the expression of metalloproteinases 2 and 9.	[31]
Temozlomide + piperine	alkaloid	U251MG T98G	in vitro	Apoptosis induction by activation of caspase-8/-9/-3, MMP loss, and inhibition of cell motility.	[32]
Temozlomide + cannabidiol	canabinoid	U87MG	in vivo mice	Controlling tumor size and improving survival.	[33]
Temozolomide + osthol	coumarin	T98G	in vitro	Apoptosis, correlated with Bcl-2/Beclin 1 complex formation.	[34]
Temozolomide + biohanin A	isoflavone	U251 U87 C6	in vitro/ in vivo rats/ in silico	Enhanced cells sensitivity to TMZ in vitro and in vivo. Inhibited TMZ-induced autophagy in GBM cells by activating the AMPK/ULK1 pathway in silico.	[35]
Temozolomide + apigenine	flavonoid	glioma cells	in vitro/ in vivo mice	Synergistic inhibition of glioma growth through the PI3K/AKT pathway.	[36]
Temozolomide + morusin	flavonoid	U87 U251	in vitro/ in vivo mice	Enhanced endoplasmic reticulum stress, synergistic effect in GBM cells, suppressed tumor progression in an orthotopic xenograft model.	[37]
Temozolomide + naringenin	flavonoid	C6 U87MG LN229 HEK-293 T	in vitro/ in vivo rats	Synergistically increased efficacy of TMZ on glioma in vitro and in vivo.	[38]
Temozolomide + xantohumol	flavonoid	U87 MG A172	in vitro	miR-4749-5p targeting RFC2 signaling participates in XN-enhanced TMZ cytotoxicity.	[39]
Temozolomide + honokiol	lignan	U87MG GL261 U87MG-R9	in vitro	Significantly enhanced TMZ-induced insults. Induced greater caspase-3 activation, DNA fragmentation, cell apoptosis, and cell-cycle arrest at the G1 phase. Autophagy and consequent apoptosis in U87-MG-R9.	[40]
Temozolomide + honokiol	lignan	U373MG GL261 U87MG	in vitro	Improved TMZ-induced insults to human malignant glioma cells. Enhanced TMZ-induced apoptosis and suppression of proliferation in human glioma cells.	[41]
Temozolomide + magnolol	lignan	LN18 U87MG LN229 T98G HEK293 C6	in vitro/ in vivo	Potentiation of TMZ-induced apoptosis in glioma by inhibiting NF-κB pathway-mediated MGMT activation.	[42]
Temozoomide + elagic acid	phenolic acid	C6	in vitro	Antiproliferative efficacy by inhibiting MGMT expression and activating apoptotic protein, p53, and caspase-3 expression.	[43]
Temozolomide + tannic acid	phenolic acid	C6	in vitro/ in vivo rats	Not cytotoxic to astrocytes. Induced anti-glioma activity, apoptosis, and cell-cycle arrest. Reduced the formation and size of colonies, and cell migration/adhesion. In vivo: decreased tumor volume and increased the area of intratumoral necrosis and infiltration of lymphocytes.	[44]
Temozolomide + elagic acid	phenolic acid	C6	in vitro	Inhibited the cadherin switch and angiogenesis.	[45]
Temozolomide + gallic acid	phenolic acid	U87MG	in vitro	Potential augmentation of the anticancer effect of TMZ via the repression of Bcl-2 expression and Akt activation and the enhancement of the p38 MAPK pathway.	[46]
Temozolomide + steroidal saponin (N45)	saponin	U87R	in vitro	Induced mitochondrial apoptosis, and decreased drug resistance by downregulation of NF-κB p65.	[47]
Temozolomide + oleuropein	secoiridoid	T98G A172	in vitro	Demonstrated additive effects that can augment the effect of TMZ.	[48]
Temozolomide + resveratrol	stilbenoid	RG-2 LN-18 LN-428	in vitro	Downregulated MGMT overexpression. Inhibition of the STAT3/Bcl-2/survivin signaling pathway.	[49]

**Table 2:** Phytocompounds or phytocompound-based products that reached the clinical trial phase (compiled based on ClinicalTrials.gov data, as of February 2024).

Compound/Product	ClinicalTrials.gov ID	Description	Study Type/Phase	Status
Cannabidiol	NCT05753007	A Clinical Trial of a Hemp-Derived, High-Cannabidiol Product for Anxiety in Glioblastoma Patients	Phase 2	Not yet recruiting
	NCT03607643	A Study of the Efficacy of Cannabidiol in Patients with Multiple Myeloma, Glioblastoma Multiforme, and GI Malignancies	Phases 1 and 2	Unknown status
TN-TC11G (Δ9-tetrahydrocannabinol + cannabidiol)	NCT03529448	TN-TC11G (THC+CBD) Combination with Temozolomide and Radiotherapy in Patients with Newly Diagnosed Glioblastoma	Phases 1 and 2	Recruiting
Cannabis (for smoking)	NCT03246113	Tolerability of Cannabis in Patients Receiving Concurrent Chemoradiation for Glioblastoma	Phase 1	Terminated
Sativex^®^ (Nabiximols oromucosal spray)	NCT01812603	A Safety Study of Sativex in Combination with Dose-Intense Temozolomide in Patients with Recurrent Glioblastoma	Phases 1 and 2	Completed
	NCT01812616	A Safety Study of Sativex Compared with Placebo (Both with Dose-Intense Temozolomide) in Recurrent Glioblastoma Patients	Phases 1 and 2	Completed
	NCT05629702	ARISTOCRAT: Blinded Trial of Temozolomide +/− Cannabinoids	Phase 2	Recruiting
Chlorogenic Acid	NCT02728349	Tolerance and Pharmacokinetic Study of Chlorogenic Acid to Advanced Glioblastoma	Phase 1	Completed
Curcumin	NCT01712542	Curcumin Bioavailability in Glioblastoma Patients	Observational	Completed
	NCT05768919	Study of Liposomal Curcumin in Combination with RT and TMZ in Patients with Newly Diagnosed High-Grade Gliomas	Phases 1 and 2	Recruiting

## Data Availability

No new data were created or analyzed in this study. Data sharing is not applicable to this article.
